# The collateral circulation determines cortical infarct volume in anterior circulation ischemic stroke

**DOI:** 10.1186/s12883-016-0722-0

**Published:** 2016-10-21

**Authors:** Estelle Seyman, Hilla Shaim, Shani Shenhar-Tsarfaty, Tali Jonash-Kimchi, Natan M. Bornstein, Hen Hallevi

**Affiliations:** 1 Stroke Unit, Department of Neurology, Tel-Aviv Sorasky Medical Center, Tel Aviv, Israel; 2 Heidelberg University School of Medicine (H.S.), Heidelberg, Germany; 3 Stroke Unit, Tel-Aviv Sorasky Medical Center, Tel Aviv, Israel; 4 Radiology Department, Tel-Aviv Sorasky Medical Center, Tel Aviv, Israel; 5 Neurology Department A, Tel-Aviv Sourasky Medical Center, Tel-Aviv, Israel

**Keywords:** Ischemic stroke, Collateral circulation, Thrombolysis

## Abstract

**Background:**

Acute ischemic stroke (AIS) is a common neurological event that causes varying degrees of disability. AIS outcome varies considerably, from complete recovery to complete loss of tissue and function. This diversity is partly explained by the compensatory ability of the collateral circulation and the ensuing cerebral flow grade.

The collateral flow to the anterior circulation largely supplies the cortical areas. The deep brain tissue is supplied by penetrating arteries and has little or no collateral supply. Although these brain compartments differ substantially in their collateral supply, there are no published data on the different effects the collateral circulation has on them. In addition, the influence of baseline collateral flow on the final infarct size following endovascular or reperfusion therapies remains unknown. This study was designed to determine the effect of the collateral circulation on cortical infarct volume and deep infarct volume, and to investigate the relation between the collateral grade, response to reperfusion therapy and clinical outcome.

**Methods:**

We studied consecutive patients presenting to our medical center between April 2008 and April 2012 with AIS and anterior proximal artery occlusion. To be included patients had to undergo a computerized tomographic angiographic study within 12 h of symptom onset demonstrating the occlusion. Imaging data and clinical and laboratory values were extracted retrospectively from the original scans and medical records. Cortical infarct volume (CIV) and deep infarct volume (DIV) were calculated as well as collateral grade. Clinical outcome was assessed at discharge using the modified Rankin Scale (mRS).

**Results:**

Of the 51 study patients, 13 were treated conservatively, 22 received intravenous recombinant tissue plasminogen activator, and 16 received intra-arterial thrombolysis. The collateral grading was similar for all three treatment groups. While there was a moderate inverse correlation between the collateral grade and CIV (Spearman’s rho = −0.49, *P* < 0.001), no comparable correlation was observed between the collateral grade and DIV (Spearman’s rho =0.03, *P* = 0.85). Clinical outcome was significantly related to CIV but not to DIV (Spearman’s rho =0.6 *P* < 0.001 versus Spearman’s rho =0.09 *P* = 0.54, respectively). The correlation between the collateral grade and CIV was greatly diminished and lost its statistical significance in patients with successful recanalization (Spearman’s *rho* = 0.2, *p* = 0.3).

**Conclusions:**

Our data shows that the collateral circulation is an important determinant of cortical infarct volume and, in turn, of clinical outcome in the setting of anterior circulation major artery occlusion. Our findings further demonstrate the benefit of recanalization in sparing cortical tissue from injury. Additional studies are needed to determine the impact of stroke therapy on the final infarct volume in relation to the collateral grade.

## Background

Acute ischemic stroke (AIS) is a common and often devastating neurologic event. It is also the second leading cause of death worldwide [1]. AIS outcome varies considerably, from complete recovery to complete loss of tissue and function. This diversity is partly explained by the compensatory ability of the collateral circulation and the ensuing cerebral blood flow [2]. The collaterals are pial arterioles connecting two major cerebral arteries that supply two different cortical territories. These arteriolar connections contribute to retrograde filling of pial arteries distal to an occlusion, and they provide alternative routes for blood flow in the setting of AIS. There is a wide inter-individual variability in size, number, and localization of the collaterals [2]. Recent evidence suggests that these collaterals are dynamic, with a time-dependent recruitment of flow to the symptomatic hemisphere, once major occlusion has occurred [3].

Although conventional angiography is considered the gold standard for collateral flow assessment [4, 5], there is wide variation in how leptomeningeal collateral grade is graded [5]. Moreover, its invasive nature makes conventional angiography impractical for first-line evaluation. Indirect assessment of collaterals can be accomplished by noninvasive methods, including transcranial Doppler (TCD), computerized tomographic angiography (CTA), and magnetic resonance angiography (MRA) [4]. Of these, CTA is the most extensively used, mostly because it is widely available, relatively noninvasive, and provides a rapid assessment of the intra- and extracranial vasculature. The independent predictive value of collaterals has been confirmed by means of different CTA methods, and inter-observer agreement within different grading scales has been shown to be acceptable [5]. Lima et al. [4] concluded that the grading of leptomeningeal collaterals on CTA is a reliable marker of good outcome in ischemic stroke and can greatly assist in the selection of patients who are potential candidates for benefitting from reperfusion therapies.

The collateral circulation may play an important role in the fate of ischemic tissue. The preservation of flow through leptomeningeal collaterals is known to reduce ischemic brain damage, especially after a proximal arterial occlusion [3, 6–12]. More recently the collateral circulation was shown to play an important role in infarct core volume [13]; and a good collateral grade was mandatory in selecting patients for intra-arterial intervention [14]. Collaterals may sustain the penumbra prior to recanalization, but the influence of baseline collateral flow on the final infarct size following endovascular or reperfusion therapies remains unknown. The collateral flow to the anterior circulation largely supplies the cortical areas. The deep brain tissue (i.e., basal ganglia and corona radiata) is supplied by penetrating arteries and has little or no collateral supply [15]. Despite these substantial vascular differences between the deep and cortical compartments, there are no published data on the differential effects the collateral circulation has after a stroke.

The purpose of our study was to determine the effect of the collateral grade on the infarct volume after an AIS involving one of the anterior circulation large arteries. We sought to differentiate between the effects of the collateral grade on the cortical infarct volume (CIV) and the deep infarct volume (DIV), and to determine the relation between the collateral grades and clinical outcome.

## Methods

The study is a retrospective analysis of imaging and clinical data. Data from the prospective Tel-Aviv Medical Center stroke registry were used to identify consecutive patients with a large artery occlusion. We screened all ischemic stroke patients who had an admission National Institute of Health Stroke Scale (NIHSS) ≥8 between 4/2008 to 4/2012. We used the NIHSS ≥8 cutoff to identify patients with a high probability of a proximal occlusion. This is slightly lower than the traditional cutoff of 10 [16], in order to increase sensitivity as was done in previous studies [17]. Inclusion criteria were having undergone a CT and a CTA within 12 h of symptom onset that demonstrated anterior circulation large artery occlusion. This was defined as an occlusion of one of the following arteries: proximal internal carotid, terminal internal carotid, proximal segment (M1) of the middle cerebral artery (MCA), and MCA bifurcation. Patients with more distal occlusions, with Anterior cerebral artery (ACA) or vertebrobasilar occlusions, and patients lacking CTA at admission or whose follow-up CT was inadequate were excluded. The medical charts were reviewed for clinical and demographic information. We used the NIHSS score to assess stroke severity and the modified Rankin Scale (mRS) on discharge as the clinical outcome measure. Decisions regarding revascularization treatment allocation or conservative treatment were made at the discretion of the attending stroke neurologist according to American Heart Association (AHA) guidelines [18]. According to these guidelines that were used at that period, the collateral circulation was not one of the determinants to choose treatment allocation.

The patients were treated either with revascularization treatment, which included: intravenous recombinant tissue plasminogen activator (IV-tPA) or intra-arterial treatment (IAT) such as clot retrievers. Intravenous thrombolysis was carried out with rt-PA (Actilyse, Boehringer Ingelheim) at a dose of 0.9 mg/kg (maximum dose 90 mg), 10 % of which as initial bolus and the remainder infused over 60 min. The Conservative therapy included antiplatelet therapy with aspirin or clopidogrel, blood pressure monitoring and hospitalization in a stroke unit. It is important to note that there were no non-pharmaceutical treatments applied, such as intravenous isotonic fluid supply or oxygenation that are considered confounding factors of collateral circulation.

The study was approved by the local Ethics Committee.

### Neuroimaging protocol

Nonenhanced CT and CTA acquisitions were performed according to standard departmental protocols with 8- or 16-section multidetector CT scanners (GE Healthcare). Nonenhanced CT was performed with the patient in a head holder in the transverse plane. CTA was performed after a 25-s delay (40 s for patients in atrial fibrillation or congestive heart failure) and administration of 100 to 140 mL of a nonionic contrast agent at an injection rate of 3 mL/s by using a power injector through an 18-gauge intravenous catheter. Images were obtained from the aortic arc up. Afterward, source images were reconstructed into standardized maximum intensity projection views of the intracranial and extracranial vasculature in the sagittal and coronal views.

### Image analysis

All CT and CTA scans were reviewed by two of the authors independently (ES and HH). Any discrepancies were resolved by a neuroradiologist (TJK). We used the General Electric Centricity® PACS RA1000 Workstation for all image analysis. The Alberta Stroke Program Evaluation of Computed Tomography (ASPECT) score [19] was calculated for each admission CT. There was no CT perfusion image analysis available for this patient cohort.

**Fig. 1:**
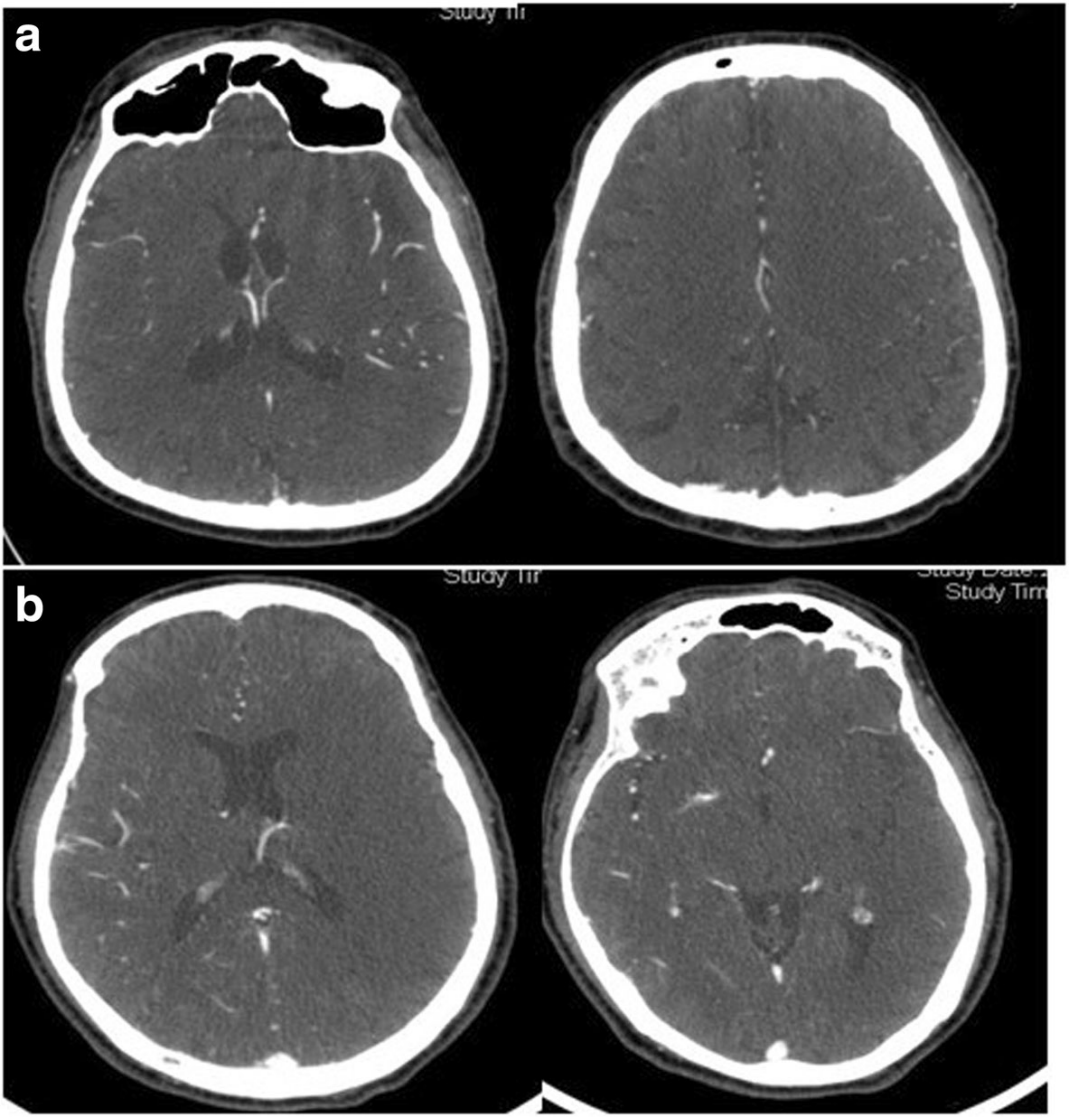
Representative CT images of the collateral circulation grading. **a** Good collateral grade (6), **b** poor collateral grade (2). Both patients presented with left MCA occlusion

The collateral circulation was graded on the CTA axial source images according to the method described by Lima et al. [4]. Briefly, the extent of vascularity was graded at two heights: around the Sylvian fissure and at the cerebral convexity as follows: 0 = absent, 1 = less than the contralateral unaffected side, 2 = equal to the contralateral unaffected side, 3 = more than the contralateral unaffected side, and 4 = exuberant. The sum of these scores is considered to be the total collateral grade ranging from 0 to 8 [3]. See Fig. 1 for representative images.

**Fig. 2:**
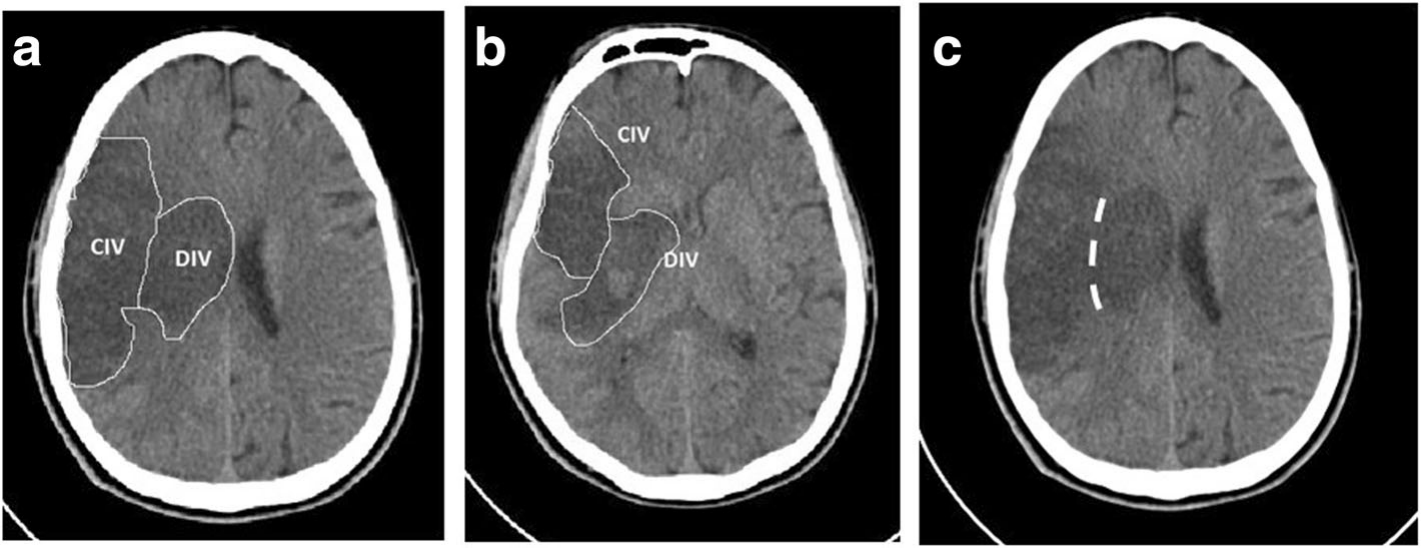
Representative CT images of the segmentation between the cortical and deep infarct areas. **a** + **b** demonstration of CIV and DIV, **c** demonstration of the natural separating line of the corona radiate. *CIV* cortical infarct volume in ml, *DIV* deep infarct volume in ml

Infarct volume was measured on a follow-up scan 24–48 h after admission by manually drawing a region of interest around the infarct margins at each slice to generate the region of interest surface area see Fig. 2 for representative images. The readers mapped both cortical and deep brain infarcts separately, using the natural separating line of the corona radiate. Each slice area was then multiplied by the slice thickness and the values were combined, taking into account slice overlap to derive the infarct volume (Fig. 2). The readers were blinded to the clinical parameter of the patients. The collateral grade, the ASPECT score and infarct volumes were read separately in order to maintain blinding.

Reperfusion was assessed only in the IAT group, when reviewing the angiographic procedure films. The score used to assess the reperfusion was Thrombolysis in Cerebral Infarction (TICI) score [20]. Good reperfusion assessed as TICI 2b-3.

### Statistical analyses

The analyses were performed using the SPSS statistics package version 21 (SPSS Inc. Chicago, IL). Spearman’s test was used to correlate the collateral grade with infarct volumes and discharge mRS. A univariate analysis of collateral predictors was carried out using the Spearman correlation for ordinal variables and the Mann–Whitney test for dichotomous variables. To reduce the likelihood of a type I error, we pre-specified the following variables to be tested: age; gender, history of hypertension, diabetes and dyslipidemia, history of heart disease, atrial fibrillation, prior stroke/transient ischemic attack (TIA), presence of carotid stenosis, aspirin and statin pretreatment, admission glucose, hemoglobin and white blood cell count, admission NIHSS, ASPECT score, time from symptom onset to CTA, stroke etiology (according to TOAST criteria) and occlusion site. Reliable information on smoking status was not available. Multiple linear regressions were used to study the interaction between the occlusion site, collateral grade and infarct volume. We assumed linear properties for the collateral grade variable for the regression model based on Harrel [21]. All tests were two-sided, and *P* values < 0.05 were considered significant.

## Results

**Fig. 3:**
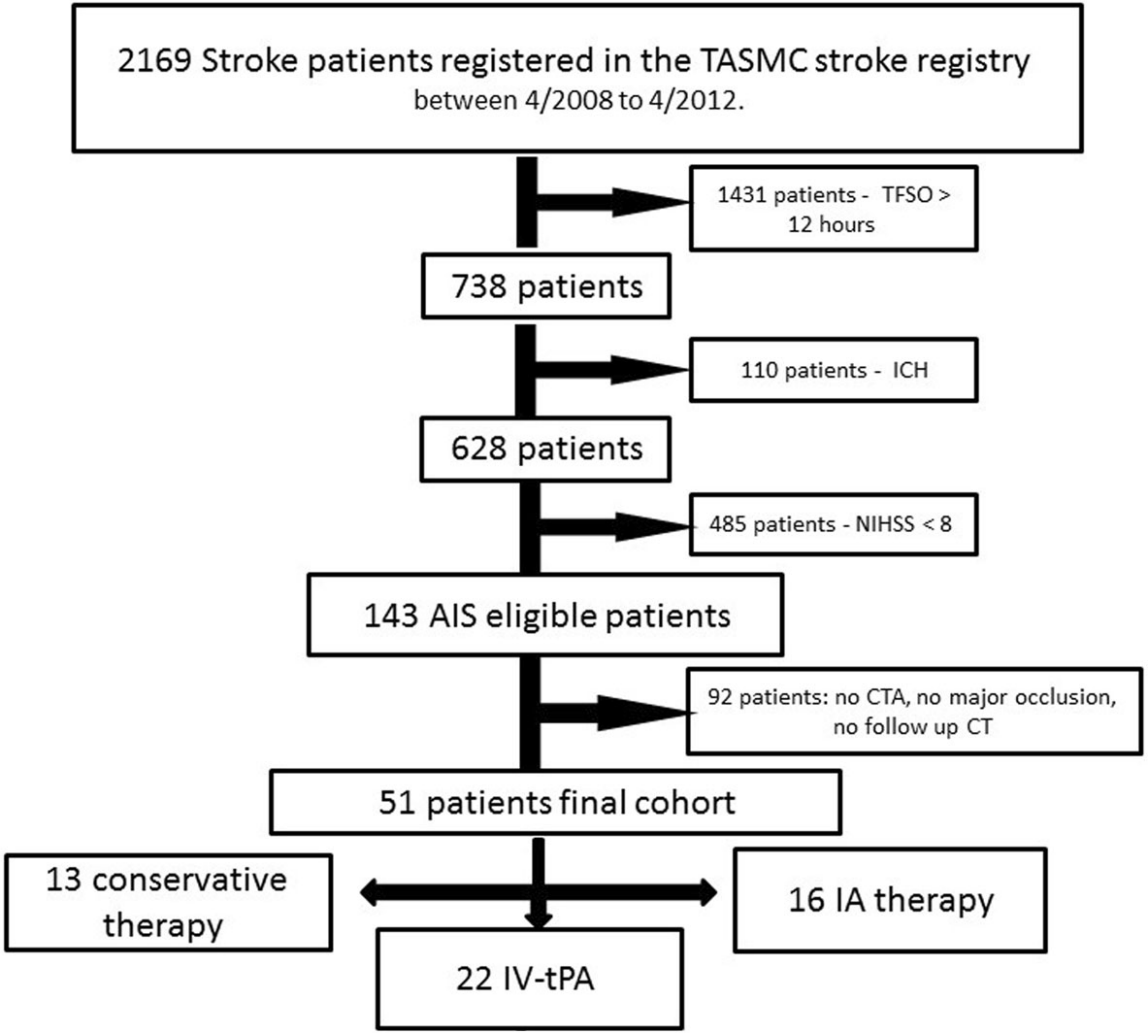
Study flowchart. *ICH* intracranial hemorrhage, *TFSO* time from symptom onset, *AIS* acute ischemic stroke, *CTA* computed tomography angiography, *IA* Intra-arterial, *TASMC* Tel-Aviv Sourasky medical center, *IV-tPA* intravenous recombinant tissue plasminogen activator

We screened a total of 2169 stroke patients from the Tel-Aviv medical center’s prospective stroke registry. Fifty-one of these patients fulfilled the study inclusion criteria (Fig. 3). The patients’ characteristics are described in Table 1. All patients were cared for by the Tel-Aviv Medical Center Stroke team. Decisions regarding revascularization treatment or conservative treatment were made at the discretion of the attending stroke neurologist. The treatment allocation was as follows: 13 patients were treated with conservative therapy (the “natural history” group), 22 were treated IV-tPA, and 16 were treated with an endovascular therapy (with or without IV tPA). The collateral grade for the three treatment groups was as follows (median, range): Conservative 4 (3–6), IV-tPA 4.5 (3–6), IAT 5 (2–8). The collateral grade for the three treatment groups was as follows (median, range): Conservative 4 (3–6), IV-tPA 4.5 (3–6), IAT 5 (2–8). The collateral grade was not statistically different within groups (*p* = 0.87, Kruskal Wallis test).

**Table 1:** Patients’ characteristics

Characteristic	*n* = 51
Age (mean ± SD)	65 ± 13
NIHSS on admission, median (range)	17 (5–25)
Collateral grading, median (range)	4 (2–8)
ASPECT score, median (range)	7 (0–10)
Time from symptoms-to-door, min (median ± IQR)	61 ± 72
Time from symptoms-to-CTA, min (median ± IQR)	113 ± 66
Parenchyma hematoma, *n*	4 (7.8 %)
Gender, *n*	
Male	25 (49 %)
Female	26 (51 %)
Site of occlusion, *n*	
Internal carotid artery - neck	5 (9.8 %)
Internal carotid artery - terminus	11 (21.6 %)
Proximal MCA	25 (49 %)
Middle cerebral artery bifurcation	10 (19.6 %)
Comorbidities, *n*	
Carotid stenosis	3 (5.9 %)
Thrombophilia	1 (2 %)
Atrial fibrillation	14 (27.5 %)
Heart disease	16 (31.4 %)
Prior stroke/transient ischemic attack	6 (11.8 %)
Dyslipidemia	16 (31.4 %)
Diabetes mellitus	13 (25.5 %)
Hypertension	31 (60.8 %)
Aspirin pretreatment, *n*	20 (39.2 %)
Statin pretreatment, *n*	20 (39.2 %)
Admission glucose, mg/dL (mean ± SD)	133 ± 47
Admission hemoglobin mg/dL, (mean ± SD)	12.8 ± 2
Admission platelets 100 k, (mean ± SD)	233 ± 79

Note: CTA indicated computerized tomographic angiography

Good reperfusion (TICI 2b-3) was achieved in six patients. The main reasons for conservative therapy were ischemic changes on admission imaging or the patient’s arrival after the designated “therapeutic window” for providing treatment. Because several patients were transferred from other hospitals, the time from symptom onset to imaging was shorter than time from symptom onset to arrival in our emergency room for 3 patients.

An inter-observer correlation analysis of the imaging data that was performed on non-study cases demonstrated an inter-rater reliability of α = 0.97 for CIV, α = 0.98 for DIV and α = 0.96 for collateral grade, all with a *P* value < 0.01.

### Correlation between collateral grade and infarct volume

**Fig. 4:**
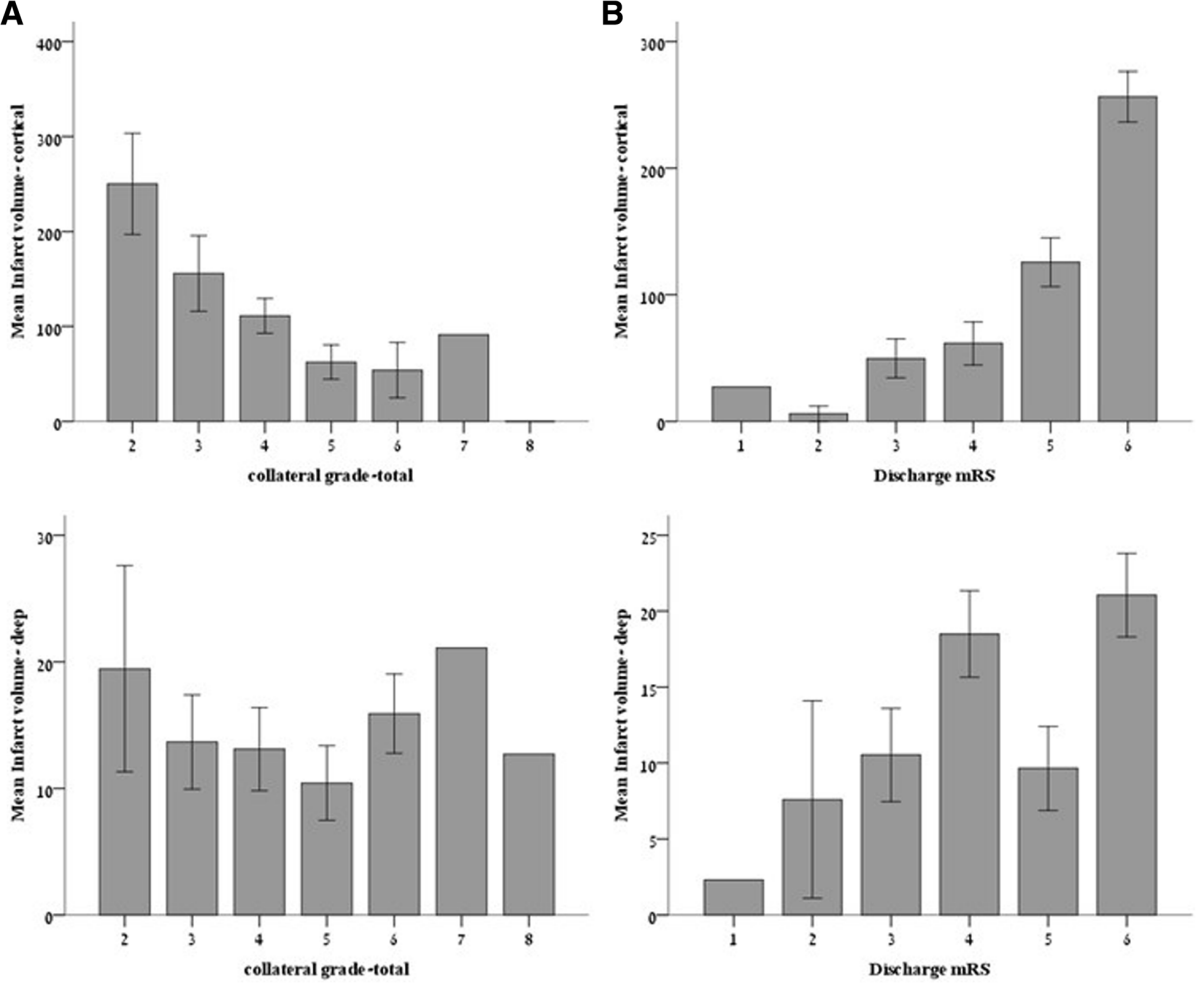
The relation of infarct volumes to collateral grade (**a**) and clinical outcome (**b**). *CIV* cortical infarct volume in ml, *DIV* deep infarct volume in ml. *Error bars* represent standard error of the mean

Collateral grade was inversely correlated with total infarct volume (Spearman’s *rho* = −0.48, *P* < 0.001). The correlations between infarct volumes in the deep and cortical compartments to collateral grade and clinical outcome are presented in Fig. 4. While there was a moderate inverse correlation between the collateral grade and CIV (Spearman’s *rho* = −0.49, *P* < 0.001), no comparable correlation was observed between the collateral grade and DIV (Spearman’s *rho* =0.03, *P* = 0.85). Similarly, clinical outcome (i.e., the mRS at discharge) was significantly related to CIV but not to DIV (Spearman’s *rho* =0.6 *P* < 0.001 versus Spearman’s *rho* =0.09 *P* = 0.54, respectively). Finally, there was a moderate correlation between the collateral grading and clinical outcome for the entire study cohort (Spearman’s *rho* = −0.41, *P* = 0.003).

Univariate analysis of the correlation between admission clinical, laboratory and radiological variables and the collateral grade revealed the following variables correlated with the collateral grade: female gender (*P* = 0.03), NIHSS (Spearman’s *rho* = −0.28, *P* = 0.04), and ASPECT score (Spearman’s *rho* =0.37, *P* = 0.007). All other tested variables did not reach a level of significance, including time parameters such as onset to CTA time or onset to treatment time. Multiple regression using these variables as predictors showed non-significant results for NIHSS and gender (*P* = 0.534 for NIHSS, *P* = 0.15 for gender), with only the ASPECT score showing a statistical trend (*P* = 0.077).

### Collateral grading cortical infarct volume

Since CIV is the main determinant of clinical outcome, and assuming that the collateral grade is not the only determinant of CIV, we performed an additional univariate and multivariate analysis on the clinical and radiological outcomes known to influence infarct volumes (NIHSS, and ASPECT score). The univariate analysis was ran after controlling for occlusion site, and revealed that all of these variables were indeed correlated with CIV (ASPECT *r* = 0.5 *p* = 0.003; NIHSS *r* = 0.48 *p* < 0.001). We next ran multiple regression analysis shown in Table 2. The R^2^ for this model was 0.39. The contribution of the collateral grade to the R^2^ of the model was 24 %.

**Table 2:** Multiple linear regression analysis of the relations between cortical infarct volume (CIV) and ASPECT score, NIHSS and collateral grade

Dependent variable	Independent variable	B	Standard error	*P* Value
CIV	ASPECT score	−5.9	4.7	0.22
	Collateral grade	−26.1	8.8	0.005
	NIHSS	6.6	2.5	0.012

### The effect of recanalization on expected cortical infarct volume

**Fig. 5:**
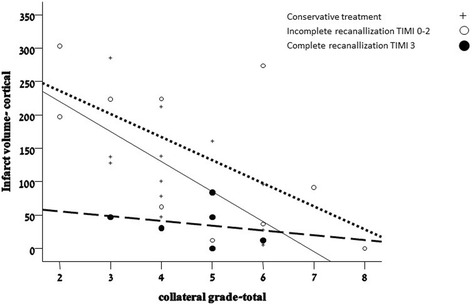
The correlation between collateral grade and cortical infarct volume according to recanalization status. *Solid line* – no recanalization therapy; *Dotted line* – TICI score = 0-2A; *Dashed line* – complete recanalization TICI score = 2B/3

Given that recanalization may be capable of sparing cortical tissue otherwise destined for infarction, the strong correlation between the collateral grade and CIV should theoretically be attenuated in patients with successful recanalization. Figure 4 depicts the association between the collateral grade and CIV according to recanalization status compared to patients that were treated conservatively: two distinct populations can be discerned based on the magnitude of the correlation between the collateral grade and CIV. The behavior of the incomplete recanalization group was identical to that of the conservative therapy group (Spearman’s *rho* =0.57, *p* = 0.04), while, in contrast, the correlation between the collateral grade and CIV was greatly diminished and lost its statistical significance in patients with successful recanalization (Spearman’s *rho* = 0.2, *p* = 0.3) (Fig. 5).

## Discussion

In this study, we analyzed the relation between three major parameters: collateral grade on admission (determined by the collateral grading scale described by Lima et al. 2010), compartmentalized infarct volume in follow-up CT imaging, and clinical outcome (mRS) at discharge. We found an inverse correlation between the total infarct volume, collateral grade and clinical outcome, in line with previous publications [10, 22].

Based on the differential vascular arrangement of the deep and cortical compartments, we predicted that better collateral circulation will protect the cortex- but not the deep compartment. Our results showed an inverse correlation of collateral grade with CIV but not with DIV, supporting this hypothesis. The strength of these correlations was significant regardless of treatment mode, stroke severity, demographic characteristics of the patients and comorbidities.

The protective effect that collaterals have on clinical outcome has already been demonstrated in previous publications [3, 23, 24], and is clearly apparent in our study cohort as well. Our results strongly suggest that the “prognostic” effect of the collateral circulation is largely mediated by its influence on the CIV.

Unlike Arsava et al. [25], we were unable to identify admission predictors of collateral grade, such as age. In fact, we were unable to predict the collateral grade based on admission variables. This underscores the importance of vascular imaging in the setting of acute stroke care. While not a substitute for penumbral imaging, the collateral grade provides a quick and reliable method of determining the degree of cortical hypoperfusion, possibly suggesting an indirect measure of imminent cortical infarction. Since current penumbral imaging has not yet become standard, nor has it been validated [26], the collateral grade may provide valuable information for the stroke clinician.

The collateral grade alone was a major predictor of cortical damage and explained 24 % of the CIV variance in the current study. We found that the NIHSS provided important additional predictive tools, almost doubling the variance explained by this model. Other factors, such as systemic blood pressure, cerebral perfusion pressure, metabolic status and recanalization [27] might further mitigate the cortical infarct volume. Measuring cerebral hemodynamic such as cerebral blood flow and mean transit time could further help predict the final infarct volumes in the different compartments.

Our results suggest that collateral grade at admission is a surrogate marker for expected cortical damage. This may no longer be the case when full recanalization is achieved; possibly indicating that recanalization spares cortical tissue otherwise destined for infarction- especially true for patients with medium collaterals (total collateral grade 3–5). This observation is supported by the recently published ESCAPE trial [14], showing that recanalization was associated with clinical benefit in patients with medium to good collaterals.

This work has several important limitations. Our findings should be regarded as hypothesis-generating only due to the study’s retrospective nature, and further validation of our results is warranted. The number of patients was small, with three different treatments applied. This limits the generalizability of our findings. The volume measurements, while accurate, were done at 24–48 h from stroke onset: this fixed time point for follow-up imaging serves for standardization, but may underestimate the final infarct volumes. Finally, our clinical outcome measure was determined on discharge. Therefore, we could not exclude the possibility that the correlation between CIV and mRS might change with longer follow-up.

In summary, our data show that the collateral circulation is an important determinant of cortical infarct volume and has little effect on the infarct volume of deep brain tissue. This in turn, profoundly affects clinical outcome in the setting of anterior circulation major artery occlusion. Our findings further demonstrate the benefit of recanalization in sparing cortical tissue from injury. Additional studies are needed to determine the impact of stroke therapy on the final infarct volume in relation to the collateral grade.

## Conclusion

The collateral circulation is an important determinant of clinical outcome after an ischemic stroke. This is modulated its selective effect on the cortical stroke volume.

## Abbreviations

AIS: Acute ischemic stroke
ASPECT: Alberta Stroke Program Evaluation of Computed Tomography score
CIV: Cortical infarct volume
CTA: Computerized tomographic angiography
DIV: Deep infarct volume
IV-tPA: Intravenous recombinant tissue plasminogen activator
MCA: Middle cerebral artery
MRA: Magnetic resonance angiography
mRS: Modified Rankin scale
NIHSS: National Institute of Health Stroke Scale
TCD: Transcranial doppler
TIA: Transient ischemic attack
